# Evaluation of Annuloaortic Ectasia by Angioscopy and IVUS “Report of 2 cases”

**DOI:** 10.1155/DTE.7.35

**Published:** 2000

**Authors:** Keiichi Tokuhiro, Yasumi Uchida, Kouhei Kawamura, Hiroshi Sakuragawa, Hiroshi Masuhara, Hidefumi Oosawa, Nobuya Koyama

**Affiliations:** Cardiovascular Center Toho University Hospital at Sakura 564-1 Shimoshizu Sakura city, Chiba 285-8741 Japan

## Abstract

We attempted combined use of angioscopy and intravascular ultrasonography (IVUS) to localize the coronary ostia and determine the aortic segment to be replaced in patients with annuloaortic ectasia, because these preoperative informations are important for selection of an appropriate technique for reconstructing the coronary artery, to prevent complications, and also to postoperative follow-up. Two cases with annuloaortic ectasia underwent angioscopy and IVUS both pre- and post-operatively. Structure of aortic cusps, position of coronary ostia, the extent of ectasia with very thin wall were clearly observed by IVUS. Angioscopy showed milky white luminal surface of the ectasic segment. After Cabrol’s operation, the sutured portion of native aorta and graft was clearly identified by IVUS and mural thrombus and naked surface of graft were observed by angioscopy. Complications were observed in none. The results indicate feasibility of combined use of angioscopy and IVUS for determination of surgical approach and follow-up in patients with AAE.


*Diagnostic and Therapeutic Endoscopy*, Vol. 7, pp. 35–45
Reprints available directly from the publisher
Photocopying permitted by license only

© 2000 OPA (Overseas Publishers Association) N.V.
Published by license under
the Harwood Academic Publishers imprint,
part of The Gordon and Breach Publishing Group.
Printed in Malaysia.

### Evaluation of Annuloaortic Ectasia by Angioscopy and IVUS "Report of 2 cases"

KEIICHI TOKUHIRO*, YASUMI UCHIDA, KOUHEI KAWAMURA, HIROSHI SAKURAGAWA, HIROSHI MASUHARA, HIDEFUMI OOSAWA and NOBUYA KOYAMA

*Cardiovascular Center, Toho University Hospital at Sakura, 564-1 Shimoshizu, Sakura City, Chiba 285-8741, Japan*

*(Received 15 May 2000; Revised 4 July 2000; In final form 4 July 2000)*

**We attempted combined use of angioscopy and intravascular ultrasonography (IVUS) to localize the coronary ostia and determine the aortic segment to be replaced in patients with annuloaortic ectasia, because these preoperative informations are important for selection of an appropriate technique for reconstructing the coronary artery, to prevent complications, and also to postoperative follow-up. Two cases with annuloaortic ectasia underwent angioscopy and IVUS both pre- and post-operatively. Structure of aortic cusps, position of coronary ostia, the extent of ectasia with very thin wall were clearly observed by IVUS. Angioscopy showed milky white luminal surface of the ectasic segment. After Cabrol's operation, the sutured portion of native aorta and graft was clearly identified by IVUS and mural thrombus and naked surface of graft were observed by angioscopy. Complications were observed in none. The results indicate feasibility of combined use of angioscopy and IVUS for determination of surgical approach and follow-up in patients with AAE.**

*Keywords*: Angioscopy, Annuloaortic ectasia, Cabrol's operation, IVUS

### INTRODUCTION

In patients with annuloaortic ectasis (AAE), identification of the extent of ectasis, sites of coronary ostia and changes of aortic valves is essential for surgical repair.

Hitherto, aortography, MRA, CT are used for evaluation of AAE. However, they give us only 2 dimentional informations. In addition, identification of fine changes in aortic wall and aortic valves is beyond their discrimination. Intravascular ultrasonography (IVUS) can discriminate a changes 0.1 mm and discriminate the changes throughout the aorta, namely, from aortic valve down to iliac artery. On the other hand, angioscopy gives us 3-dimentional macroscopic pathological informations on aortic luminal surface. Therefore, we attempted the combined use of angioscopy and IVUS for evaluation of AAE before and after surgery.

* Corresponding author. Tel.: +81-43-462-8811. Fax: +81-43-463-1456. E-mail: tokuhiro@med.toho-u.ac.jp.

### MATERIALS AND METHODS

### Angioscopy

Fiberscopes with an outer diameter of 0.8 1.4 mm were connected to an illumination source (Olympus) and CCD camera. The obtained pictures were recorded on S-VHS recorder simultaneously with angioscopic images and were displayed on a monitor. A balloon-tipped 9Fr guiding catheter was used to guide the angioscope. The tip of the catheter can be bent to form a J shape and the bending angle can also be adjusted using a bending device. Moreover, this system enables bull's eye observation of the target lesions, and does not require a large amount of physiological saline for displacement of blood. To observe a target lesion, the guide wire was introduced under fluoroscopic guidance, and a balloon guiding catheter was introduced to the target lesion. Then, the angioscope was advanced and fixed at the most distal tip of the guiding catheter. The balloon was inflated with $CO_2$ and pushed against the wall. Physiological saline containing heparin was injected by a power injector for displacement of blood. Injection speed of physiological saline was 2 to 3 ml/sec, and the observation period was up to 10 seconds. Observation was repeated when necessary.

### Intravascular Ultrasonography (IVUS)

IVUS probe was 9Fr. in external diameter, 20 MHz 30 rps, mechanical sector (Olympus) and monorail type. The probe was introduced to the aortic cusp and then gradually pulled back to observe the lesions.

### PATIENTS AND OPERATIVE PROCEDURES

In this study, 2 patients with AAE underwent angioscopy and IVUS. Neither of them had Marfan syndrome.

Case 1 was a 61-year-old male with a past history of esophageal tumor. He had been followed for aortic regurgitation since 1993. After aortography, angioscopy and IVUS, he underwent surgery because the ascending aorta was dilated and mitral regurgitation progressed. He did not have Marfan somatotype, and had chest X-P CTR of 50%. ECG showed atrial fibrillation. Surface ultrasonography showed moderate mitral regurgitation and advanced aortic regurgitation. The maximum diameter of the ascending aorta was 60 mm and the diameter of the aortic valve ring was 24 mm. Aortic regurgitation was classified into Class III of the Sellers' classification. The dilated ascending aorta was replaced with a composite graft consisting of a Hemashield 28 mm artificial vessel and SJM 25 mm artificial valve with continuous retrograde cardioplegia under mild hypothermia. The Cabrol technique with a Hemashield 8 mm artificial vessel was selected for reconstructing the coronary artery. Annuloplasty with a 34 mm Carpentier-Edwards ring was performed for the mitral valve. The aortic cross clamp time was 3 hours and 12 minutes and a pump-oxygenator was used for 4 hours and 20 minutes. The postoperative course was uneventful, and the patient was discharged in good condition on Day 33 postoperatively. He underwent aortic angiography, IVUS, and angioscopy 1.5 months after discharge.

Case 2 was a 48-year-old female with a complaint of breathlessness. She did not have Marfan somatotype, and had chest X-P CTR of 61%. Surface ultrasonography showed that the diameter of the aortic valve ring was 21 mm and that the maximum diameter of the ascending aorta was 65 mm. No marked arteriosclerotic findings were noted. Aortic angiography which was performed with angioscopy and IVUS, showed aortic regurgitation (AR) (Class III of the Sellers' classification) with a pear-like aortic root. Surgery was chosen because the enlargement of the ascending aorta progressed in addition to advanced AR due to enlarged aortic valve ring. Perioperative observation showed that the aorta had a diameter of 70 mm and was enlarged for 10 cm from the base. The affected site was replaced with a composite graft consisting of a SJM 21 mm HP artificial valve and 26 mm Hemashield artificial vessel with continuous retrograde cardioplegia under mild hypothermia. The Cabrol type anastomosis with an 8 mm Hemashield artificial vessel was used in reconstructing the coronary artery. The aortic cross clamp time was 2 hours and 33 minutes and a pump-oxygenator was used for 3 hours and 17 minutes. The postoperative course was uneventful, and the patient was discharged in good condition on Day 20 postoperatively. She again underwent angiography, angioscopy, and IVUS to follow the affectedsite 1 month after discharge.

### RESULTS

Fig. 1(A) shows a preoperative aortic angiogram of Case 1. The whole Valsalva sinus was dilated. Aortic regurgitation was Class III. A thinned vascular wall could be confirmed by IVUS. Fig. 1(B) and (C) show an IVUS image of the dilated segment and a partically enlarged distal aorta, respectively.

Calcified plaque was observed at the 7:00 o'clock direction. This thickening was too small to be detected by angiography or surface ultrasonography.

Fig. 2 shows preoperative angioscopic images of Case 1. The ecstatic segment (Fig. 2(A)) was white in color. Fig. 2(B) image shows ascending aorta. No marked arteriosclerotic lesions were noted in ectatic and neighboring non-ectatic segments.

Fig. 3(A) shows a preoperative aortic angiogram of Case 2. A so-called pear-like dilation of the aorta was observed mainly in the right Valsalva sinus. Aortic regurgitation was classified III. The IVUS image of the arrowed site in Fig. 3 (B) shows smooth and diffusely thinned wall. Fig. 3(C) shows an enlarged portion. The wall thickness was an approximately 2 mm and luminal surface was uneven.

Fig. 4 (A: systolic phase), (B: diastolic phase) shows IVUS images of the preoperative aortic valve ring, and coaptation failure of the cusps. The left coronary artery could be found on the left and the left main trunk coronary artery could be clearly identified, as shown in Fig. 4(C).

Fig. 5(A) shows a preoperative angioscopic image of the dilated ascending aorta of Case 2. The aortic intima was smooth and white. Fig. 5(B) is an image just peripheral to the dilated segment. This portion was smooth and had yellow arteriosclerotic plaques.

A B C

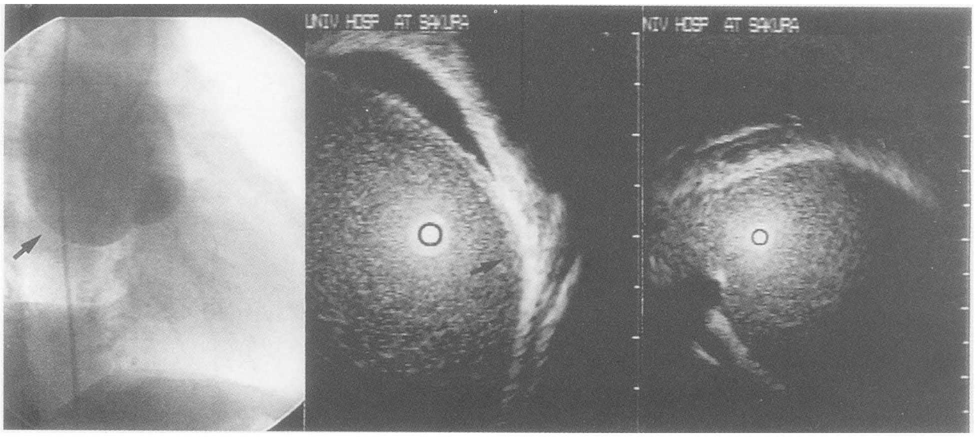

FIGURE 1 Preoperative aortic angiogram and IVUS images of Case 1. A. Dilated valsalva sinus and class III aortic regurgitation (arrow). B. Thin wall of the dilated portion (arrow). C. A partially enlarged distal ascending aorta. (Calcified plaque at 7:00 o'clock direction).

A B

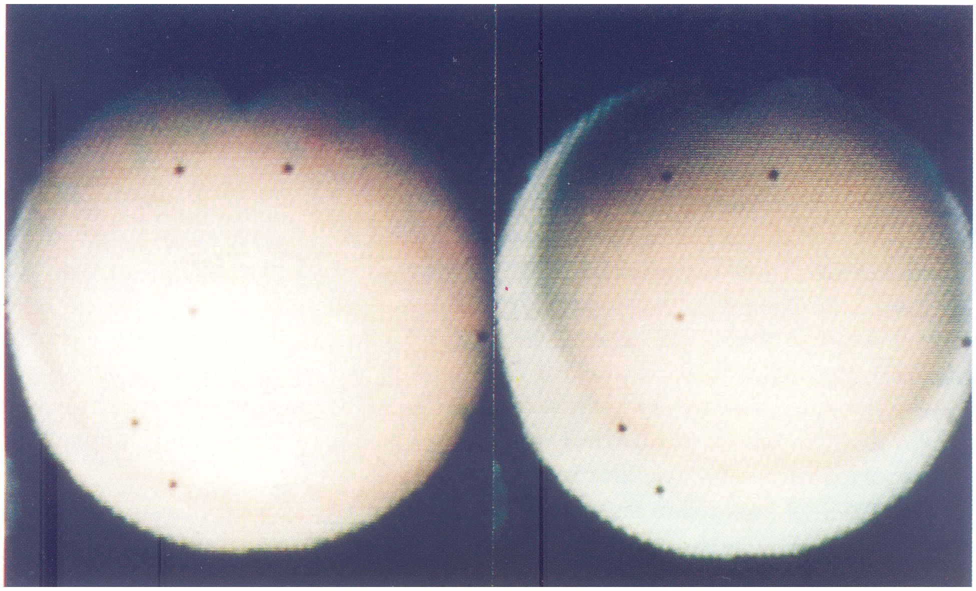

FIGURE 2 Preoperative angioscopic images of Case 1. A. Milky white luminal surface of the ectatic portion. B. White distal non-ectatic ascending aorta.

A B C

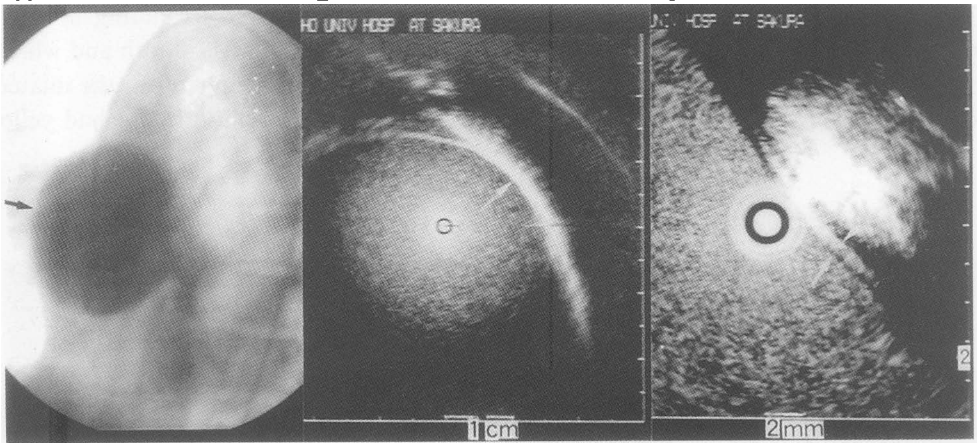

FIGURE 3 Preoperative aortic angiogram and IVUS images of Case 2. A. Pear-like dilation of the aorta and class III aortic regurgitation (arrow). B. IVUS images of thinned ectatic segment (arrow). C. Magnified feature of the same segment (arrow: 2 mm-thick wall).

### (After operation)

Fig. 6(A) shows an aortic angiogram of Case 1 at 1.5 months postoperatively. The artificial vessel connected to the ascending aorta was too long and was bended. However, the artificial valve was normally functioning. By IVUS, the peripheral anastomosis between the artificial vessel and native aorta was not stenotic and anastomotic failure was not observed (Fig. 6(B)).

A B C

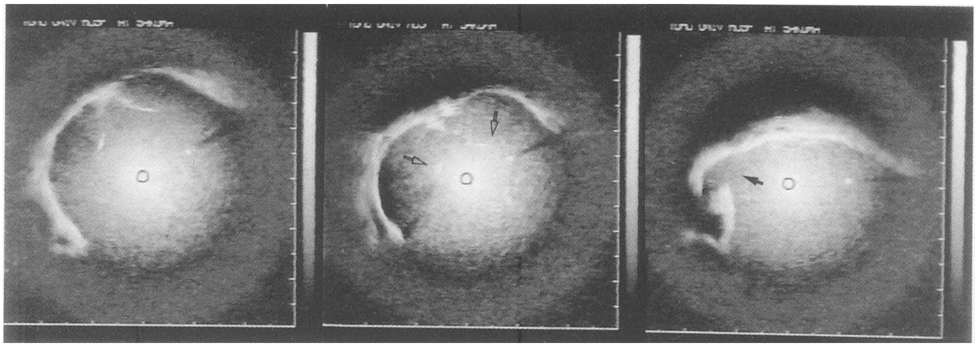

FIGURE 4 Preoperative shorter axis IVUS images of Case 2. A. Systolic phase at aortic valve level B. Diastolic phase at aortic valve level. Coaptation failure (arrow). C. Sinus Valsalva and left main trunk of coronary artery.

A B

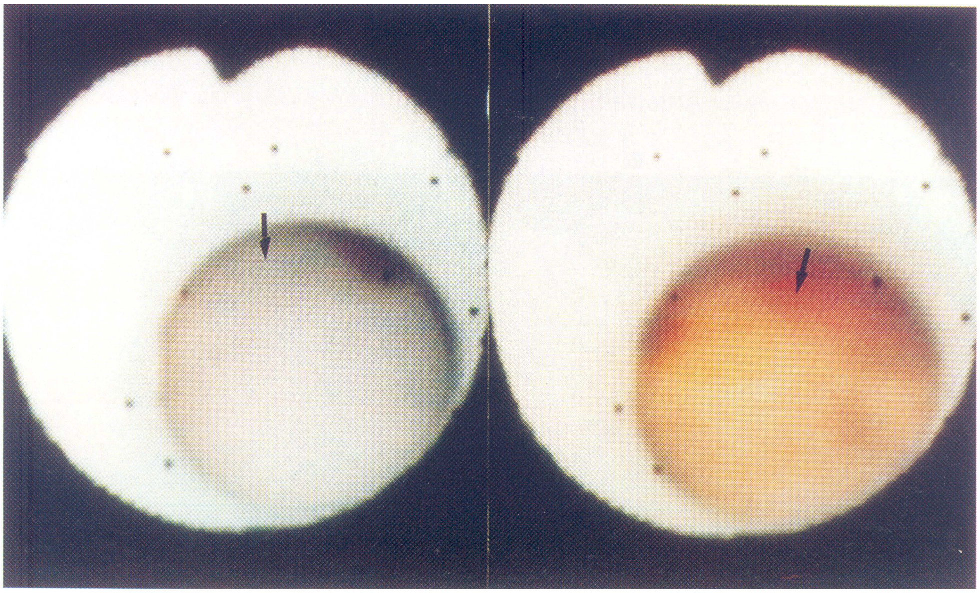

FIGURE 5 Preoperative angioscopic images of Case 2. A. White surface at elastic segment. B. Yellow arteriosclerotic plaques in distal non-ectatic ascending aorta.

Fig. 6(C) shows an IVUS image of the artificial vessel itself. The bended part of the artificial vessel was clearly observed.

Fig. 7 shows a postoperative angioscopic image of Case 1. Panel (A) shows a mural red thrombus.

Panel (B), the luminal surface of the artificial vessel was uncovered with endothelium. The mesh of the artificial vessel was clearly observed. No thrombus was observed, Panel (C) corresponds to bended portion of the artificial vessel.

Histological examination revealed disruption of elastic fibers (Fig. 8(A),(B)), and cystic medial necrosis with acid mucopolysaccharide accumulation (Fig. 8(A),(B)).

Also, waving and disruption of collagen fibers typical to AAE were observed (Fig. 9(A),(B)).

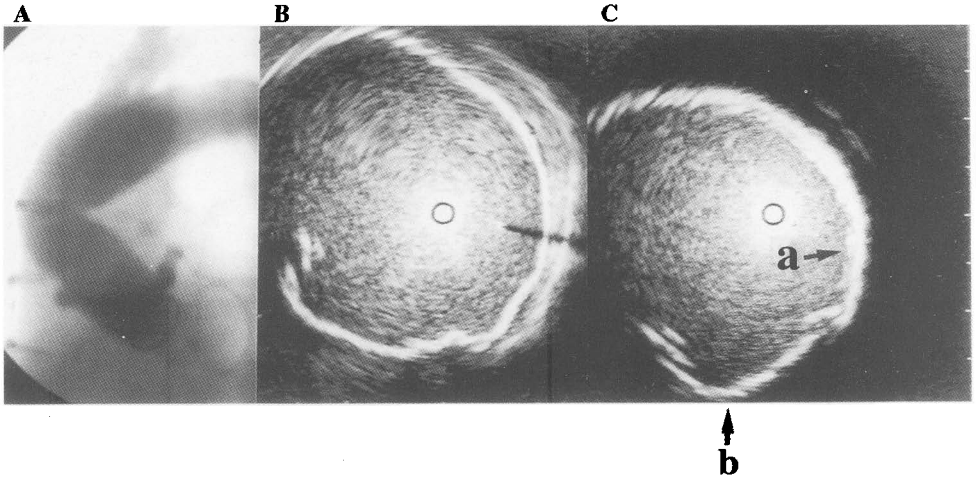

FIGURE 6 Postoperative (1.5 months later) aortic angiogram and IVUS images of Case 1. A. The artificial vessel. B. Distal of artificial vessel (no stenosis and no thrombus). C. IVUS image of the distal anastomosed site (arrow a; native aorta, arrow b; graft).

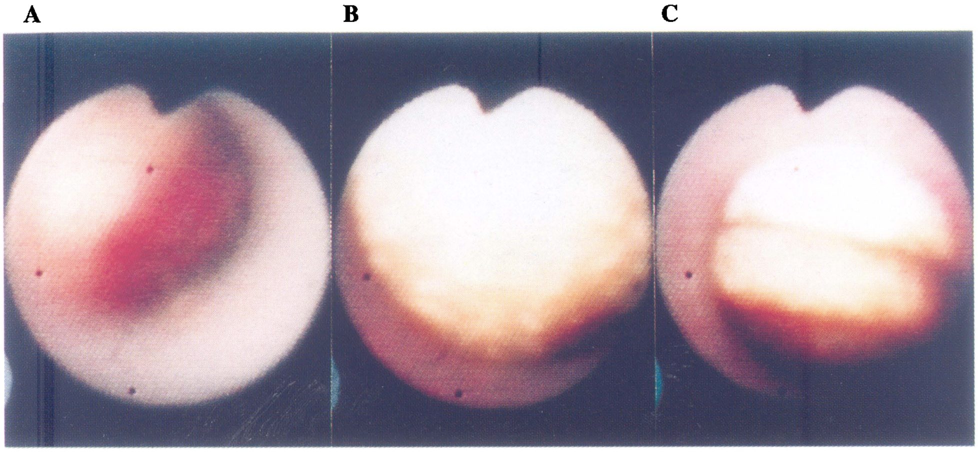

FIGURE 7 Postoperative angioscopic images of Case 1. A. Mural thrombi on the graft. B. Luminal surface of the artificial vessel, with naked mesh. C. Multiple folds at the bent artificial vessel.

IVUS of removed ecstatic segment revealed thin media (Fig. 10(A)) and scanning microscopy revealed waving intimal surface (Fig. 10(B)). After Evans blue dye staining, wavy surface became more clearly visible (Fig. 10(C)).

In case 2, waving of collagen fibers and disruption were also observed (Fig. 11(A), (B)).

### DISCUSSION

The patients with AAE are usually asymptomatic unless aortic dissection cardiac failure by aortic regurgitation develops. However, once these complications develop, AAE often progresses rapidly. Although no standard has been established for the

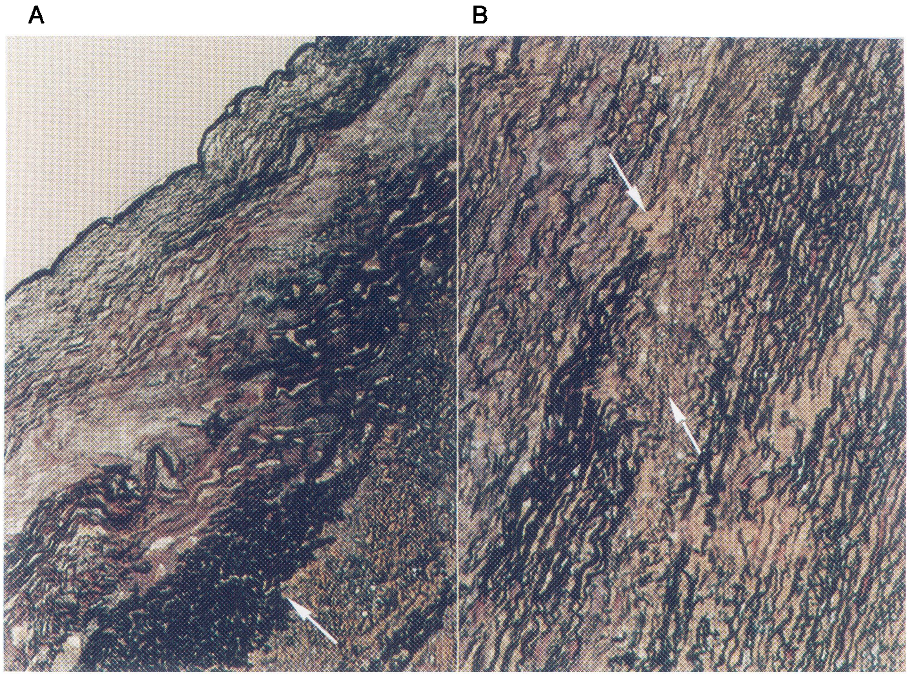

FIGURE 8 Histological changes in the ectatic segment on Case 1. (Elastica Van Gieson stain × 200). A. Fragmentation of elastic fibers and Asid mucopolysaccharide accumulation at black spot (white arrow). B. Disruption of elastic fibers (white arrow).

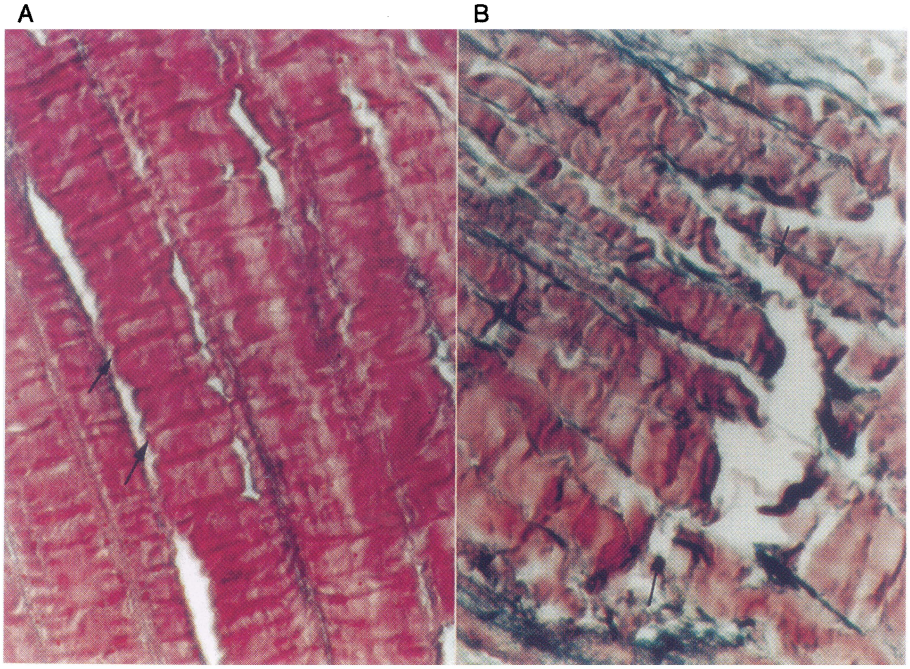

FIGURE 9 Histological findings of the aortic wall in Case 1. (Reticulin silver impregnation × 200). A. Waving of collagen fibers (arrows). B. Disruption of collagen fibers (arrow).

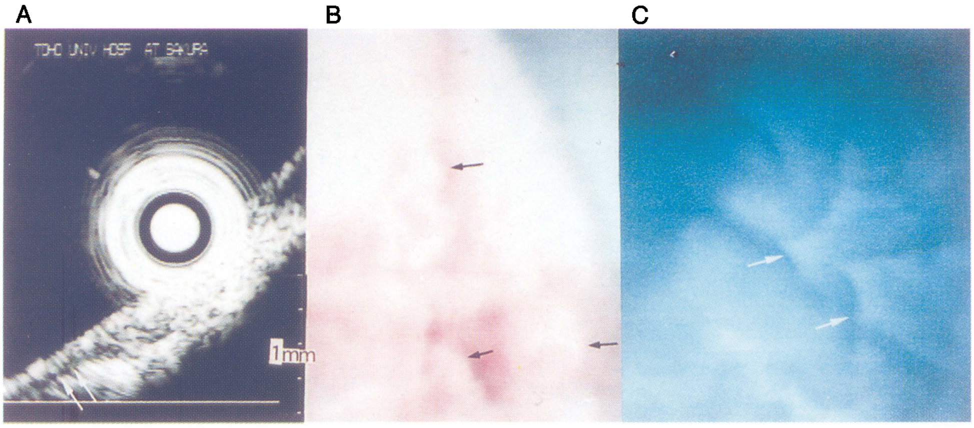

FIGURE 10 Removed of ectatic segment of Case 2. A. IVUS image. B. Thin media. Wavy luminal surface. (Microscope). C. Evans-blue stain of the same portion. Wavy changes. (Microscope).

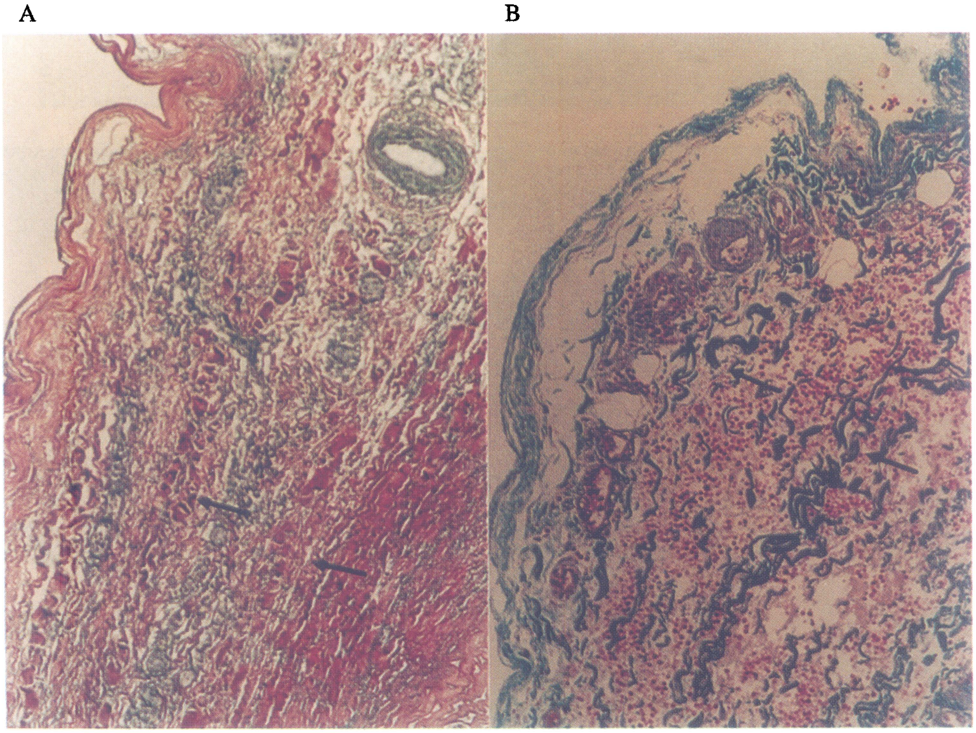

FIGURE 11 Histological changes of the ectatic segment in Case 2. A. Waving of collagen fibers. (Reticulin silver impregnation × 200). B. Rupture of elastic fibers. (Elastica Van Gieson stain × 200).

treatment of AAE, medical therapy is generally chosen when the aortic diameter is less than 6 cm and surgery is chosen when it is 6 cm or more. Surgery is usually chosen even when the aortic diameter is less than 6 cm if AAE is advanced and complicated with serious cardiac failure or if

AAE causes serious myocardial ischemia [1]. Recent surgical outcomes indicate 7-year survival of 75% in those with AAE [2] and 9-year survival of 82% in those with Marfan syndrome [3]. The prognosis of AAE is good if its complications are surgically prevented. Therefore, accurately determining its disease stage is important not only in early detection, but also in medical follow-up, and the disease stage is the key for determining the time to switch from medical to surgical therapy. The surgical techniques for AAE have been improved and been applied even to elderly patients. Moreover, an aortic valve-sparing operation that can reduce the complications of anti-coagulant therapy has been clinically applied. Due to these therapeutic changes, it has become necessary to fully evaluate the aortic valve, coronary arteries and ascending aorta pre- or peri-operatively. The angiographic informations required for evaluating the aortic wall and Valsalva sinus are often insufficient due to insufficient opacification with contrast material. In some cases, coronary angiography cannot be performed because the dislocation of the coronary ostia hinders the insertion of a catheter. Since the surgery for AAE requires reimplantation of the coronary artery, it is desirable to collect accurate informations on the coronary ostia and its surroundings. Furukawa *et al.* [4] reported that perioperative evaluation of the aortic valve is important for aortic valvuroplasty and they angioscopically observe the aortic valve during the cardiac standstill. They have also reported that direct observation of the aortic valve before aortic declamp is considered to be useful for prediction of the aortic valve function after valvuloplasty. However, since this evaluation prolongs the clamping time of the aorta, it would be better to avoid taking the time for perioperative examinations. Therefore, we attempted the use of angioscopy and IVUS for evaluating AAE preoperatively.

Although angioscopy was established in the 1980's, its application as a diagnostic measure was gradual. It could not gain popularity because the blood in the target vessel had to be excluded and replaced with a translucent liquid or the fiber had to be thick to keep its resolution [5]. However, it has increasingly been used for clarifying coronary lesions and other smaller vessels. Aortic angioscopy for aortic surgery was applied clinically by Uchida *et al.* and is now routinely used clinically [6]. Angioscopes become smaller and its resolution power was much improved. IVUS is useful for evaluation of wall structure and therefore for determining the degree of vascular stenosis. With angioscope, it is impossible to examine every detail of arterial intima within limited time. Therefore, the following measures were taken to observe the target. Firstly, the observational part was decided by IVUS and fluoroscopic image. Secondly the angioscope was guided either by J-shape cathetel with remote controllable bending angle or by cathetel with various shaped balloons. Finally, while pulling back the cathetel, the approximate target could be observed.

In our AAE cases preoperatively, angioscopy gives us intimal surface informations. The aorta intima appears smooth and milky white color in ectatic segment. At non-ectatic segment, the aorta was dotted with yellow atherosclerotic plaques that were not detected in IVUS.

After operation, the luminal surface of the artificial vessel was observed. Almost surface was not uncovered with endothelial tissue and mesh of the artificial vessel was clear naked surface in postoperative acute phase. But mural red thrombus, fresh thrombi, was detected in neighboring anastomosis. Pulsatile blood flow was observed in the wrinkle of the bended artificial vessel. These results suggest that preventive measures against thrombosis may be required.

Since IVUS provides a cross-sectional image of a vessel, it can clearly represent the three-layered structure of the arterial and aortic wall. Therefore, it is useful for evaluating the histologic properties. In surgery, intimal hyperplasia determines the patency of the anastomosis of an auto- or artificial vessel. As an intravascular arteriosclerotic stenotic lesion, intimal hyperplasia is involved in the stenosis or thrombotic formation at the anastomosis. To investigate this problem, the anastomosis of an artificial vessel with aortic tissue was observed with angioscopy and IVUS in our two cases. As preoperative result of IVUS, conditions of aortic valve, structure of aortic wall, especially the thin and calcified legion and arterial branch were confirmed.

The confirmation contributed to acknowledgement of aortic valve working in ill status, information of aortic cross clamp, selection of surgical procedure e.g. incisional line and prevention of complication.

As preoperative result of angioscopy, both 3-dimentional pictures in sections and macroscopic pathological information of intima were acquired. The acquisition helped to prevent complication caused by aortic injury, rupture of arteriosclerotic plaque. It also contributed, similarly with IVUS, to the decision of surgicial procedure e.g. incisional line, excisional section. Atherosclerotic change in arterial intima can be judged to some extent by its color and property. Uchida *et al.* [7] make classifications from the difference in color change that is observed through angioscopic appearance of coronary plaque. As a result of pathological correlations, especially the glistening yellow plaques are unstable plaque, and the plaque shows similar color change in the case of aorta.

This time again, we conducted the systematic examination of tissue sample and evaluation of macroscopic information, retrospectively. In addition, regarding thrombus formative parts, the old and new of thrombus can be judged from its color.

After the operation of aortic disease, the key result was the anastomotic information acquired by both IVUS and angioscopy. With angioscope, blood flowing in the wrinkle made in the artificial vessel and formation of mural red thrombus were observed. In addition, confirmed were status of bending artificial vessel and its projecting edge against intima. Intimal structure is normally hard to observe by means of angiography and conventional cardiac. Therefore, attained information of intimal structure is valuable for consideration of surgical procedures, observation of intima of anastomosis in progress and drug control of anticoagulant therapy.

Fortunately, intimal hyperplasia or abnormality was not observed at the anastomosis probably because it was observed in the postoperative acute stage. However, micro-thrombi possible due to the inflammatory change at the anastomosis were observed. In this case, the thrombus confirmed after the operation was micro thrombi. Therefore, it could be coped by merely increasing the anticoagulant. However, if the thrombus formation had been larger, additional methods such as mechanical hydrodynamic thrombectomy or thrombolysis therapy would have been necessary. In our two cases, surgery was performed with Cabrol's technique, in which coronary blood flow is clearly different from physiological blood flow. It has been confirmed that coronary blood flows spirally when Cabrol's technique is used. It has been reported that coronary blood flows in a whirlpool manner at a stenotic site and that thrombi are formed in a doughnut manner [8]. In future, it may be necessary to analyze the blood flow of the Bentall's modification with angioscopy and IVUS when it is used to reconstruct the coronary artery.

Gunen *et al.* [9] examined intimal hyperplasia associated with experimental vascular anastomosis with angioscopy and IVUS, and reported that the sensitivity in identifying 550 $\mu$ m-thick intimal hyperplasia was 88% for angioscopy and 100% for IVUS. This suggests the predominance of IVUS in identifying intimal hyperplasia. AAE is different from other arteriosclerotic changes, such as aneurysm, in that it is quite fragile. The markedly thinned arterial wall of AAE has been supported by IVUS findings. Histological examination has also shown typical disruption of elastic fibers. Halme *et al.* [10] have reported that many AAE patients showed reduced elastin concentration and increased collagen concentration in the dilated aortic wall, and suggested that the biochemical heterogeneity of the aortic wall may influence the onset of AAE. In patients with Marfan syndrome, the concentrations of elastin and collagen in the aortic wall are almost normal when it is not macroscopically dilated, while elastin concentration is reduced when the aortic wall is dilated. This reduction of elasting may be the cause of the white appearance of AAE as observed with angioscopy.

AAE patients develop some arteriosclerotic lesions with aging. However, their arteriosclerotic lesions have been reported to be more elastic than normal ones and close to normal tissue in perioperative observation. In our case, yellow atherosclerotic change on luminal surface was softy and nearly white color in several places.

It has also been reported that their lesions histologically show reduced elastin concentration and increased collagen concentration. Since these changes in color are observed, the pathologic examination of angioscopic images of AAE may be switched from macroscopic to microscopic examination of tissue properties in future.

Thus, angioscopy and IVUS were used in combination for selection of an appropriate surgical technique. Examinations as to whether to apply them to less-invasive stent graft delivery surgery are now underway [11]. Hill *et al.* [12] experimentally delivered a stent to an aortic target lesion by blocking its central side with a balloon. They reported that the stent could be more accurately delivered with angioscopy than fluoroscopy because the former provides a direct view.

Angioscopy does not have the problem of parallax often associated with fluoroscopy because it provides three-dimensional images. Angioscopy is also advantageous in that it does not require radiation exposure and contrast medium injection. Therefore, it can eliminate possible risk in some patients, such as allergic reaction and nephropathy. IVUS provides information on the arterial diameter and the thickness of the arterial wall, but has a limitation that it provides no information while a device, such as a stent graft, is delivered. Thus angioscopy and IVUS in combination gives no much more information on aortic changes in AAE and for selection of therapeutic modalities otherwise not obtainable.

### References

[1] Onodera, K. and Fujino, Y. Annuloaortic ectasia *Ryoikibetsu Shokogun.* 1996; **13**: 268–272.

[2] Niederhauser, U., Kunzli, A. and Genoni, M., *et al.* Composite graft replacement of the aortic root: long-term results, incidence of reoperations. *Thorac. Cardiovasc. Surg.* 1999; **47**: 317–321.

[3] Niinami, H., Aomi, S. and Tagusari, O., *et al.* Extensive Aortic Reconstruction for Aortic Aneurysms in Marfan Syndrome. *Ann. Thorac. Surg.* 1999; **67**: 1864–1867.

[4] Furukawa, K., Higuchi, S. and Ueno, T., *et al.* An aortic valve-sparing operation (David's operation) for a patient with annulo-aortic ectasia (AAE) and aortic regurgitation (AR) – evaluation by intraoperative endoscopy. *Nippon Kyobu Geka Gakkai Zasshi.* 1996; **44**: 826–829.

[5] Tomaru, T. and Kobayakawa, N. Angioscopy and intravascular ultrasound. *Nippon Rinsho.* 1999; **57**: 1531–1536.

[6] Uchida, Y. Cardioangioscopy *Tokyo: Medical View* 1995; 38–43.

[7] Uchida, Y., Kanai, M. and Takeuchi, K., *et al.* Angioscoptic appearance of vulenrable coronary plaque, and it's pathological correlations. *Coronary* 1999; **16**: 302–313.

[8] Uchida, Y., Nakamura, F. and Tomaru, T., *et al.* Rheological significance of tandem lesion of the coronary artery. *Heart Vessels* 1995; **10**: 106–110.

[9] Gunen, E., Trubel, W. and Schwarzacher, S., *et al.* Evaluation of distal anastomotic intimal hyperplasia: an experimental comparison by arteriography, duplex sonography, angioscopy, and intravascular ultrasound. *Eur. Surg. Res.* 1999; **31**: 64–73.

[10] Halme, T., Savunen, T. and Aho, H., *et al.* Elastin and collagen in the aortic wall: changes in the Marfan sysndrome and annloaortic ectasia. *Exp. Mol. Pathol.*, 1985; **43**: 1–12.

[11] Hill, B., Neville, R. and Hyde, G., *et al.* Angioscopic evaluation of an endoluminal aortic graft: the first clinical experience. *J. Endovasc. Surg.* 1995; **2**: 248–254.

[12] Hill, B.B., Hyde, G.L. and Kuo, C.S., *et al.* Aortoscopy: a guidance system for endoluminal aortic surgery. *J. Vasc. Surg.* 1996; **24**: 439–447.

